# Multifunctional elastin-like polypeptide renders β-glucosidase enzyme phase transition and high stability

**DOI:** 10.1186/s13068-019-1497-5

**Published:** 2019-06-24

**Authors:** Yang Zhou, Xiaofeng Li, Dandan Yan, Frank Addai Peprah, Xingqi Ji, Emmanuella Esi Fletcher, Yanwei Wang, Yingying Wang, Jie Gu, Feng Lin, Haifeng Shi

**Affiliations:** 1 Institute of Life Sciences, Jiangsu University, No. 301 Xuefu Road, Zhenjiang, 212013 People’s Republic of China; 2 Key Laboratory of Healthy Freshwater Aquaculture, Ministry of Agriculture, Zhejiang Institute of Freshwater Fisheries, Huzhou, 313001 People’s Republic of China

**Keywords:** *Coptotermes formosanus*, Termites, β-Glucosidase, Glycosyl hydrolases, Elastin-like polypeptides, Protein purification, Protein stability, Biofuels

## Abstract

**Background:**

In the enzymatic conversion of biomass, it becomes an important issue to efficiently and cost-effectively degrade cellulose into fermentable glucose. β-Glucosidase (Bgluc), an essential member of cellulases, plays a critical role in cellulosic biomass degradation. The difficulty in improving the stability of Bgluc has been a bottleneck in the enzyme-dependent cellulose degradation. The traditional method of protein purification, however, leads to higher production cost and a decrease in activity. To simplify and efficiently purify Bgluc with modified special properties, Bgluc-tagged ELP and His with defined phase transitions was designed to facilitate the process.

**Results:**

Here, a novel binary ELP and His tag was fused with Bgluc from termite *Coptotermes formosanus* to construct a Bgluc–linker–ELP–His recombinant fusion protein (BglucLEH). The recombinant plasmid Bgluc expressing a His tag (BglucH) was also constructed. The BglucLEH and BglucH were expressed in *E. coli* BL21 and purified using inverse transition cycling (ITC) or Ni-NTA resin. The optimum salt concentration for the ITC purification of BglucLEH was 0.5 M (NH_4_)_2_SO_4_ and the specific activity of BglucLEH purified by ITC was 75.5 U/mg for substrate *p*-NPG, which was slightly higher than that of BglucLEH purified by Ni-NTA (68.2 U/mg). The recovery rate and purification fold of BglucLEH purified by ITC and Ni-NTA were 77.8%, 79.1% and 12.60, 11.60, respectively. The results indicated that purification with ITC was superior to the traditional Ni-NTA. The *K*_m_ of BglucLEH and BglucH for *p*-NPG was 5.27 and 5.73 mM, respectively. The *K*_ca_*t*/*K*_m_ (14.79 S^−1^ mM^−1^) of BglucLEH was higher than that of BglucH (12.10 S^−1^ mM^−1^). The effects of ELP tag on the enzyme activity, secondary structure and protein stability were also studied. The results showed that ELP tag did not affect the secondary structure or enzyme activity of Bgluc. More importantly, ELP improved the protein stability in harsh conditions such as heating and exposure to denaturant.

**Conclusion:**

The Bgluc–linker–ELP–His system shows wide application prospect in maintaining the activity, efficient purification and improving the stability of Bgluc. These properties of BglucLEH make it an interesting tool to reduce cost, to improve the efficiency of biocatalyst and potentially to enhance the degradation of lignocellulosic biomass.

**Electronic supplementary material:**

The online version of this article (10.1186/s13068-019-1497-5) contains supplementary material, which is available to authorized users.

## Background

Lignocellulose, mainly comprising of cellulose (20–50%), hemicelluloses (15–35%) and lignin (18–35%), is the most abundant biomass on earth and is recognized as a potential sustainable source for biofuels. Cellulose is a polysaccharide composed of long linear chains of d-glucose linked by β-(1,4) glycosidic bonds [1]. Enzymatic hydrolysis of cellulose requires the synergistic action of endoglucanases (EG, EC 3.2.1.4), cellobiohydrolases (CBH, EC 3.2.1.91), and β-glucosidases (Bgluc, EC 3.2.1.21) [2]. The CBH acts on the ends of the cellulose chain and releases β-cellobiose as the end product; EG randomly attacks the internal O-glycosidic bonds, resulting in glucan chains of different lengths; and the Bgluc catalyzes the hydrolysis of β-glycosidic bond in glucosyl derivatives like aryl-glucosides, cellobiose, or higher cellooligosaccharides to produce glucose. Bgluc themselves are also subject to product inhibition by glucose [3].

These enzymes are members of the glycoside hydrolases family (GHF) with hydrolyze the glycosidic bonds between carbohydrates. Termites have developed cellulose digestion capabilities with endogenous and symbiotic cellulases, with the endogenous cellulolytic enzymes consisting of EG and Bgluc [4]. This dual-cellulose digestion system appears to result in high digestibility of cellulose. The lower termites, *Coptotermes formosanus*, are often nicknamed the super-termite because of its destructive habits due to the large size of its colonies and its ability to degrade lignocellulosic polysaccharides efficiently at a rapid rate [5]. Based on the amino acid sequences, Bglucs have been classified into GHF 1, 3, 5, 9, 30 and 116 [6]. In termite-derived endogenous cellulases, EGs are affiliated with the GHF9; while all endogenous Bglucs belong to GHF1, except for one putative endogenous GHF3 Bgluc [1].

Bgluc plays a vital role in cellulose hydrolysis by undertaking the rate-limiting final step of hydrolyzing cellobiose, which is an intermediate product of cellulose hydrolysis and also a strong inhibitor of cellulase activities [7]. It is a common practice to supplement Bgluc to increase the efficiency of cellulose saccharification. The insufficiency of Bgluc in this cellulase complex is one of the bottlenecks in efficient cellulose hydrolysis [8]. To efficiently digest the cellulase in biomass, certain issues need to be addressed and resolved. First, much effort needs to be taken to improve the activity of Bgluc, or a wide variety of Bgluc can be acquired at lower cost or at best to utilize effective methods such as recombinant enzyme. Second, the traditional column chromatography purification has some disadvantages, such as been time-consuming, irreversible adsorption and low reproducibility, which calls for the need to develop an inexpensive, productive and simple purification procedure. Third, to increase the utilization of enzymes, enzyme engineering needs to focus on improving stability and decreasing the loss of activity in harsh conditions such as heating and exposure to denaturant [9]. However, there is a lack of an integrated system for the production, efficient purification, and techniques for improving Bgluc stability for its large-scale application as a biocatalyst for biomass conversion into bioethanol.

Elastin-like polypeptides (ELPs) are repetitive artificial polypeptides derived from recurring amino acid sequences found in the hydrophobic domain of tropoelastin [10]. The most commonly used ELP has the pentapeptide sequence (VPGXG)_*n*_, where the guest residue X can be occupied by any amino acid except proline (because proline destroys the inverse phase transition property of the ELP), and “*n*” represents the number of pentapeptide repeats in the ELP [11]. ELPs exhibit a quick and thermodynamic reversible phase transition behavior at a specific temperature referred to as the inverse transition temperature (*T*_t_) [12, 13]. ELPs are structurally disordered and soluble in aqueous solution below their *T*_t_; whereas, intramolecular contacts between the nonpolar regions result in aggregation, and hence precipitation above the T_*t*_ [14]. This transition of ELP is a reversible process, so the ELP can be fully resolubilized when the solution temperature is below the *T*_t_. The transition of ELPs and their fusion proteins can also be isothermally triggered by reducing the *T*_t_ below solution temperature by the addition of salts from the Hofmeister [15].

The inverse transition cycling (ITC) provides a new and efficient strategy for protein purification. This technique can be used to achieve a rapid, high protein purity using the thermally triggered phase-separation behavior of ELP to enable a simple separation and purification of their fusion proteins [11, 16]. In this study, a gene from *C. formosanus* encoding Bgluc tagged with the novel multifunctional binary protein, ELP&His (Bgluc–Linker–ELP–6xHis, BglucLEH) was synthesized. Furthermore, the recombinant plasmid expressing the fusion protein Bgluc with a His tagged (BglucH) was also constructed. To achieve biological activity, a short peptide linker ((GGGGS)_3_) was inserted between the Bgluc and the ELP. The recombinant BglucLEH and BglucH were cloned into pET-28a(+), and expressed in the *E. coli* BL21. BglucLEH was purified with ITC and Ni-NTA resin separately, whereas BglucH was purified with only Ni-NTA resin. We evaluated the purification efficiency by comparing BglucLEH purified by ITC with BglucLEH and BglucH purified by Ni-NTA resin. Finally, ELP labeling effects on the kinetic parameters, enzyme activity and stability were also studied.

## Results

### The design of fusion protein (BglucLEH and BglucH) and the construction of recombinant expressional plasmid

**Fig. 1:**
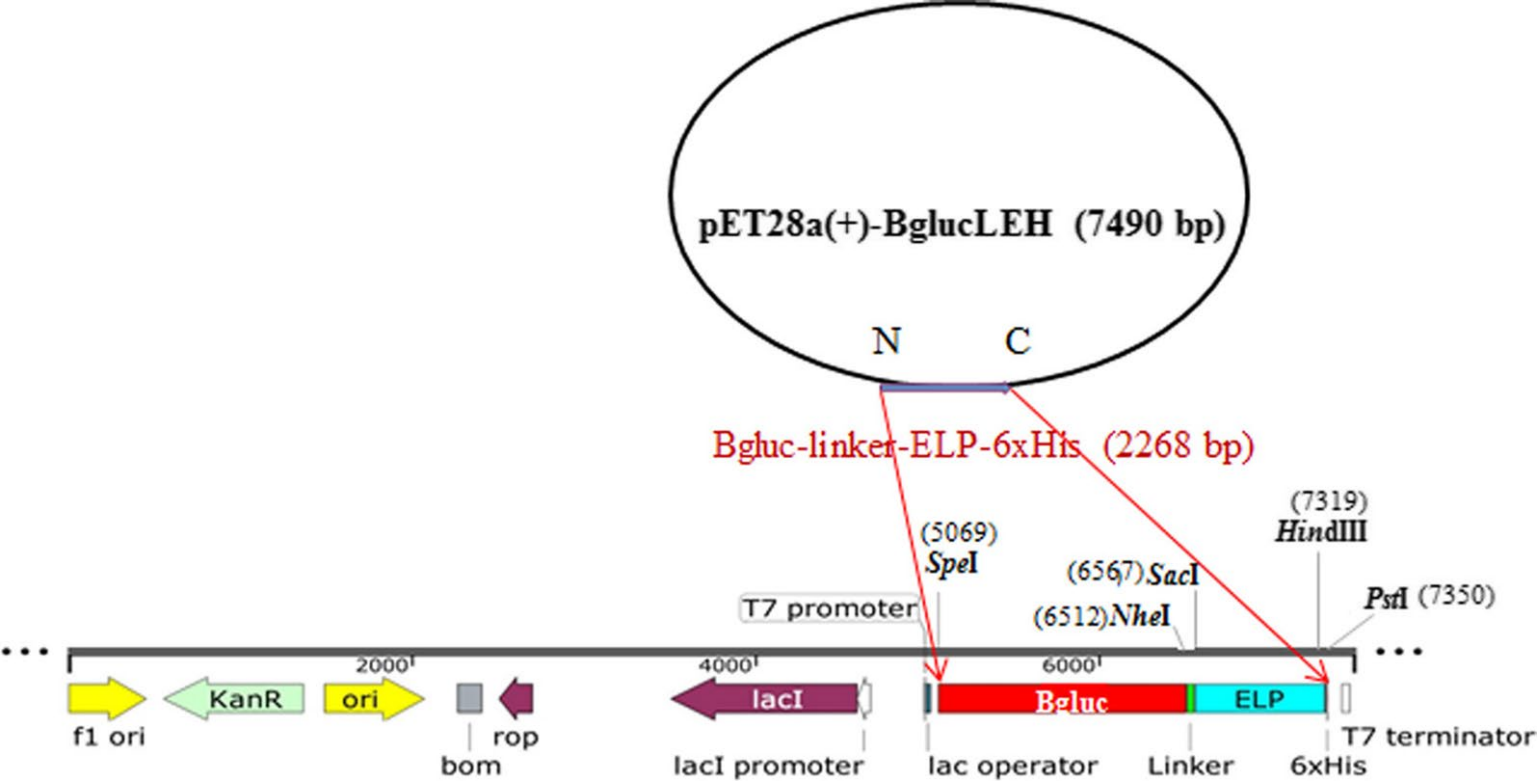
Schematic diagram of recombinant plasmid pET28a(+)-BglucLEH

Bgluc from the termite *C. formosanus*, a 479-amino acid enzyme, was encoded in a 1437-bp DNA fragment. The length of the DNA fragments encoding linker, ELP and His was 45, 750 and 18 bp, respectively, as shown in Fig. 1. The length of the DNA fragments of BglucLEH was 2268 bp. Furthermore, the recombinant plasmid pET28a(+)-Bgluc (BglucH) containing His-tagged Bglu was previously constructed [5]. The recombinant plasmids were verified by DNA sequencing.

### Production of BglucH and BglucLEH

The molecular weights of BglucH and BglucLEH are around 56 and 82 kDa, respectively. SDS–PAGE analysis of the soluble and insoluble fractions following expression in *E. coli* revealed that BglucH and BglucLEH accumulated primarily in an insoluble fraction at 37 °C. The induction of protein expression at lower temperatures increases the yield of soluble recombinant proteins in *E. coli* [17, 18]. To obtain active soluble proteins, we induced the expression of BglucH and BglucLEH at 25 °C with 1 mM isopropyl-beta-d-thiogalactopyranoside (IPTG) (Additional file 1: Figure. S1a), and the result showed that BglucH and BglucLEH were produced in soluble form (Additional file 1: Figure. S1b).

### Purification of BglucH and BglucLEH

**Fig. 2:**
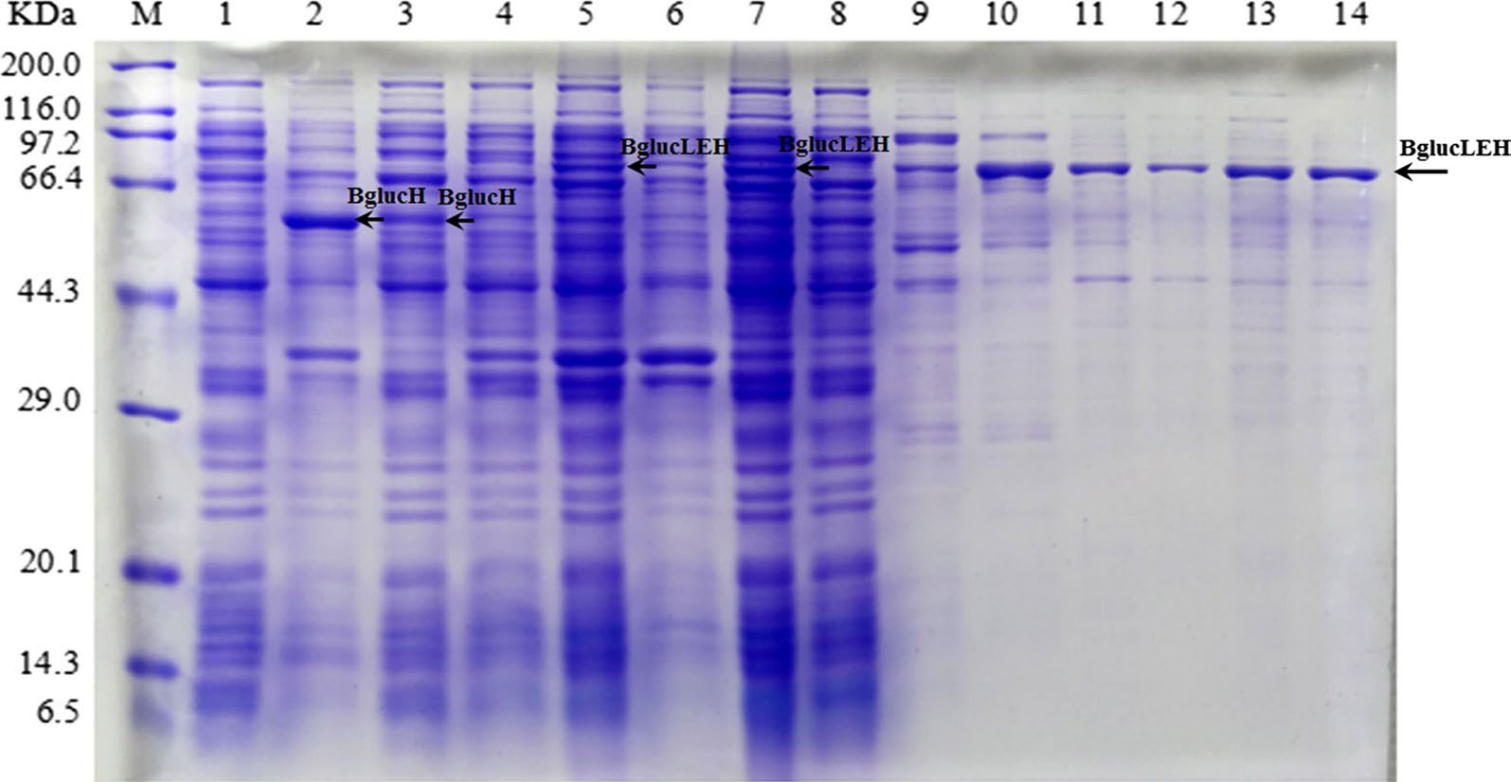
SDS–PAGE analysis of BglucLEH production and purification by Ni-NTA resin and ITC. Lane M: protein molecular weight marker (Broad); lane 1: crude cell lysate of cell with empty pET-28a(+) vector; lane 2: insoluble part of crude lysates containing BglucH; lane 3: crude cell lysate containing BglucH; lane 4: cell lysates of cell with BglucLEH plasmid without IPTG induction; lane 5: crude lysates of cells with BglucLEH expression; lane 6: insoluble part of crude lysates of cells with BglucLEH expression; lane 7: crude cell lysate containing BglucLEH; lanes 8, 9, 10: eluted BglucLEH with 20, 100, 400 mM imidazole, respectively; lanes 11, 12, 13, 14: BglucLEH purified by ITC with 1 M NaH_2_PO_4_, 1 M (NH_4_)_2_SO_4_, 1 M Na_2_SO_4_ or 2.5 M NaCl, respectively

**Fig. 3:**
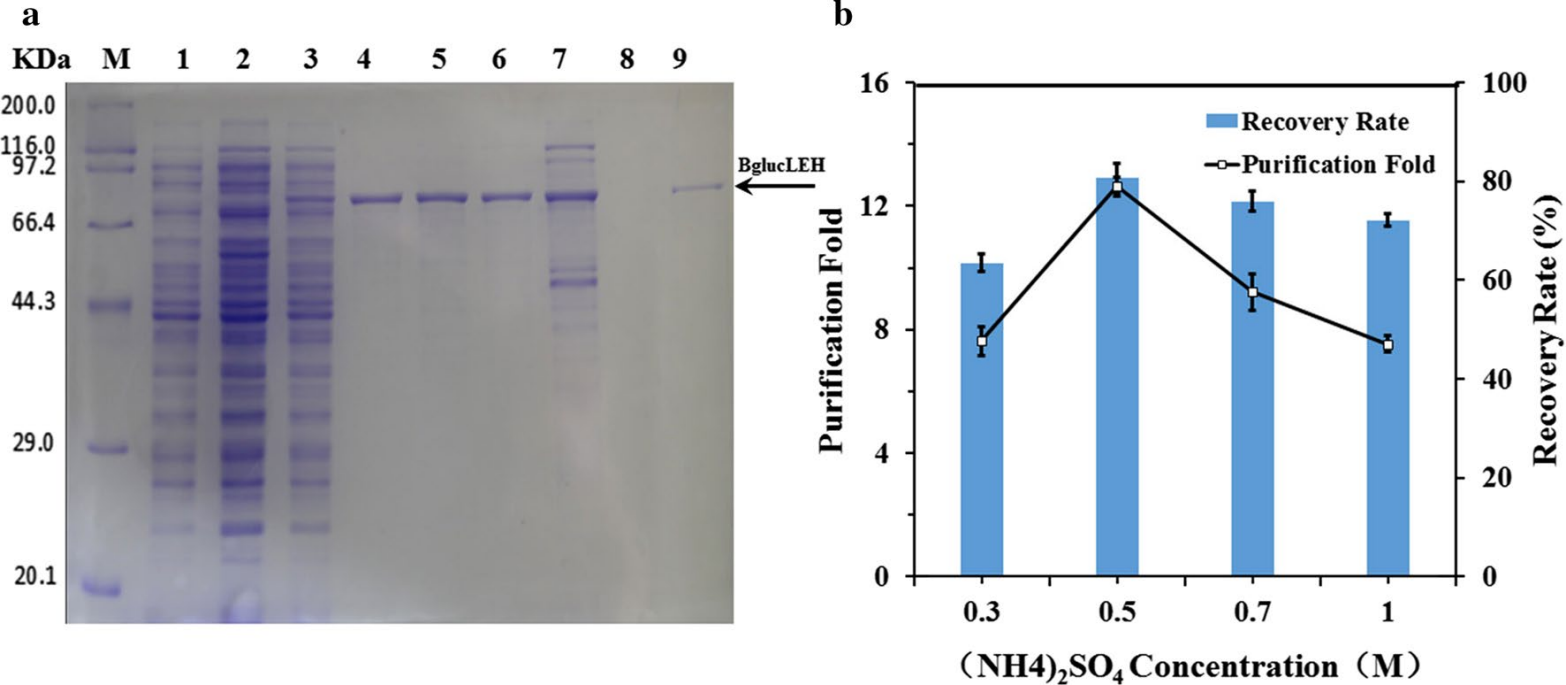
SDS–PAGE of fusion proteins BglucLEH purified by ITC method (**a**). Lane M: protein molecular weight marker (Broad); lane 1: crude lysate of cells with empty pET-28a(+) vector; lane 2: crude lysate of cells with BglucH expression; lane 3: crude lysate of cell with BglucLEH expression; lanes 4–7: purified BglucLEH by ITC using various (NH_4_)_2_SO_4_ concentrations (0.3, 0.5, 0.7, 1.0 M); lane 8: little proteins purified from crude lysate containing BglucH by ITC using 0.5 M of (NH_4_)_2_SO_4_; lane 9: BglucLEH purified by Ni-NTA resin. Purification efficiency by ITC method using various (NH4)_2_SO_4_ concentration (**b**)

The effect of Hofmeister series ions on the *T*_t_ is given as: NH_4_^+^ > K^+^ > Na^+^ > Mg^2+^ > Ca^2+^ > Mn^2+^ > Cu^2+^; SO_4_^2−^ > HPO_4_^2−^ > CH_3_COO^−^ > Cl^−^ > Br^−^ > NO_3_^−^ > SCN^−^. According to the series, 1 M NaH_2_PO_4_, 1 M (NH_4_)_2_SO_4_, 1 M Na_2_SO_4_, 2.5 M NaCl were selected to purify the BglucLEH. The expression levels and purity of collected fractions were determined by SDS–PAGE (Fig. 2, Lane 11–14). According to the above result (Fig. 2), (NH_4_)_2_SO_4_ was selected as precipitant and the effect of the various concentrations of (NH_4_)_2_SO_4_ (0.1, 0.3, 0.5, 0.7, 1.0 M) on the purification efficiency was investigated. A clear band at size around 82 kDa appeared obviously indicating that 0.3–0.7 M (NH_4_)_2_SO_4_ could be used to purify BglucLEH by ITC (Fig. 3a, lane 4, 5, 6). Meanwhile, it can be seen from Fig. 3b that the purification fold reached a maximum value of 13.3 at 0.5 M (NH_4_)_2_SO_4_. With the increase of (NH_4_)_2_SO_4_ concentration from 0.7 M to 1.0 M, the purification fold decreased gradually. The recovery rate of the BglucLEH reached the maximum value (80%) at 0.5 M (NH_4_)_2_SO_4_. Thus, 0.5 M (NH_4_)_2_SO_4_ was identified as the optimum precipitant for the purification of BglucLEH.

The purification of BglucLEH was conducted utilizing two techniques, which are Ni-NTA resin and ITC method because BglucLEH contains His and ELP tags (Fig. 2), and the two methods are independent of each other. BglucH, however. was purified using only Ni-NTA resin because it contains only a His tag (Additional file 1: Figure S2). To compare the ITC with Ni-NTA resin, the purification efficiency of BglucLEH either by ITC or Ni-NTA resin, and that of BglucH by Ni-NTA resin are summarized in Table 1. The specific activity of BglucLEH by ITC was 75.50 U/mg, which was slightly higher than that of BglucLEH by Ni-NTA resin (68.22 U/mg) (*p *< 0.01) and BglucH by Ni-NTA resin (68.85 U/mg) (*p *< 0.01). The recovery rate and purification fold of the BglucLEH by either ITC or Ni-NTA resin were 77.78%, 79.07% and 12.60, 11.39, respectively, and the recovery rate and purification fold of the BglucH by Ni-NTA resin were 75.62% and 12.14, respectively. Furthermore, the densitometric analysis of SDS–PAGE indicated that the purity of BglucLEH by one-round ITC reached 90%, which showed an excellent performance in the purification process (Fig. 3a, lane 5).

**Table 1:** A comparison of purification efficiency between Ni-NTA resin and ITC

Proteins	Total protein (mg)	Specific activity (unit/mg)	Total activity (unit)	Yield (%)	Purification (fold)
Cell lysate^a^ (all proteins of *E. coli contained EP*)	3.25 ± 0.39	0.08 ± 0.004	0.263 ± 0.06		
Cell lysate with expression of BglucH^b^(all proteins of *E. coli* contained BglucH)	6.63 ± 0.53	5.75 ± 0.042	37.59 ± 0.89		
BglucH^b^ in cell lysate	6.63 ± 0.53	5.67 ± 0.35*	37.33 ± 0.78	100.0	1.0
BglucLEH^c^ in cell lysate (all proteins of *E. coli* contained BglucLEH)	5.23 ± 0.34	6.07 ± 0.46	31.32 ± 0.65		
BglucLEH^c^ in cell lysate	5.23 ± 0.34	5.99 ± 0.54*	31.06 ± 0.63	100.0	1.0
Purified BglucH by Ni-NTA resin	0.41 ± 0.09	68.85 ± 1.19	28.23 ± 0.51	75.62	12.14
Purified BglucLEH by Ni-NTA resin	0.36 ± 0.04	68.22 ± 1.27	24.56 ± 0.59	79.07	11.39
Purified BglucLEH by one round of ITC	0.32 ± 0.05	75.50 ± 1.54	24.16 ± 0.68	77.78	12.60
Purified BglucLEH by two rounds of ITC	0.27 ± 0.03	76.22 ± 1.43	20.58 ± 0.47	66.26	12.72

*EP* empty plasmid pET-28a(+)

^a,b,c^2.5 mL cell extracts was obtained through ultrasonication treatment of *E. coli* collected from 75 mL LB culture

* Indicates that the specific activity of the empty carrier has been removed

Furthermore, the effect of the number of ITC on the purification was also investigated. The specific activity of BglucLEH by one- and two-round ITC was 75.50 U/mg and 76.22 U/mg, respectively (Table 1). The recovery rate and purification fold of the BglucLEH by one- and two-round ITC were 77.8%, 66.26% and 12.60, 12.72, respectively (Table 1). The densitometric analysis of SDS–PAGE showed that the purity of BglucLEH by two rounds of ITC did not significantly increase compared with a single round of ITC (Additional file 1: Figure S3), but decreased the recovery rate (Table 1).

### ELP tag did not change the secondary structure of Bgluc

**Fig. 4:**
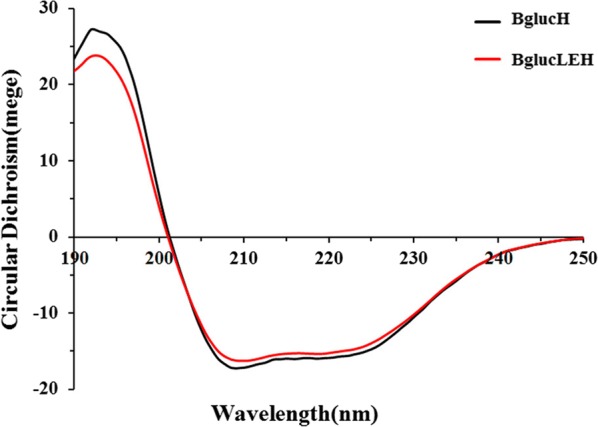
The circular dichroism spectrum of BglucH and BglucLEH

To explore the secondary structure of Bgluc in the presence of ELP, circular dichroism (CD) analysis for BglucH and BglucLEH was performed from 190 to 250 nm. The result showed one positive peak around 196 nm and two negative peaks trough around 208 nm and 222 nm, indicating the typical signature for β-sheet and α-helix structure (Fig. 4), respectively. Analysis of the secondary structure propensities for BglucH and BglucLEH from the results showed no significant structure difference (Fig. 4). This indicated that the ELP tag appears to have little effect on the secondary structure of Bgluc.

### Optimum pH and temperatures of BglucH and BglucLEH

**Fig. 5:**
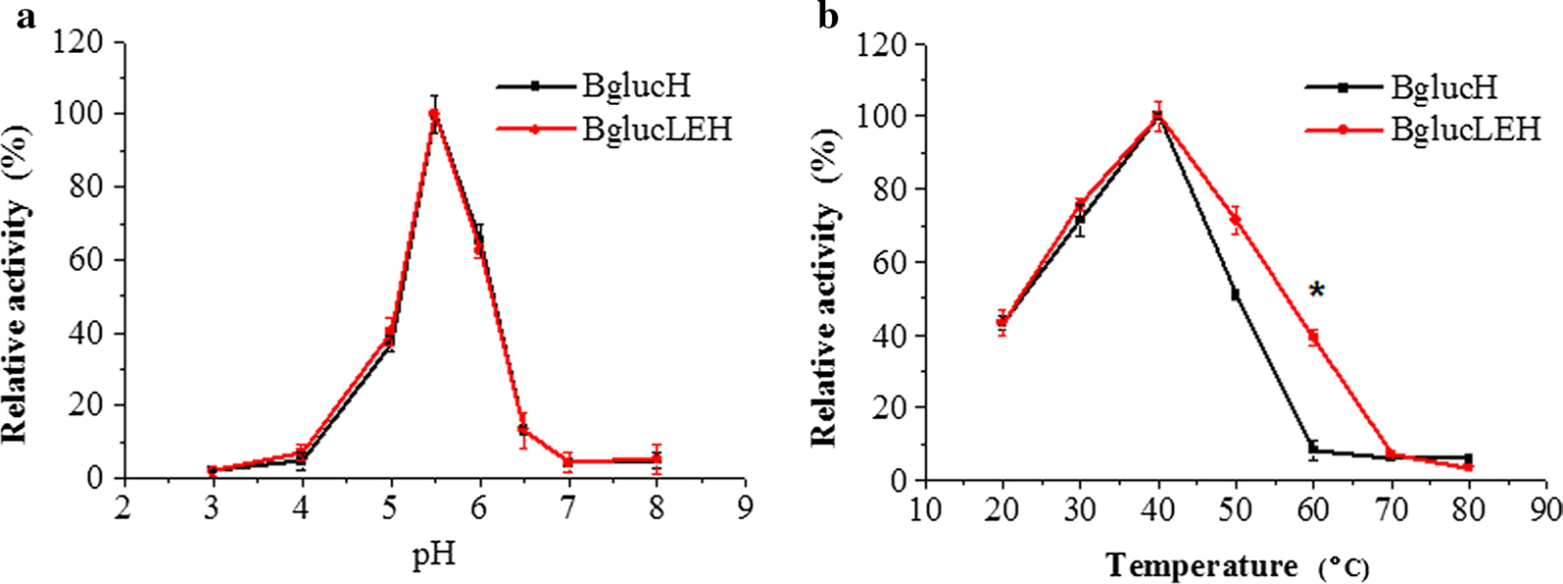
Effects of pH (**a**) and temperature (**b**) on the enzyme activity. Each value in the panel represents the mean ± SD (*n* = 3). Asterisks denote statistical significance. *p < 0.05, **p < 0.01

The influence of temperature and pH on the activities of BglucLEH was studied compared with BglucH. It was observed that both BglucH and BglucLEH had their highest activities at optimum pH of 5.5 (Fig. 5a), which indicates that ELP tag did not change the optimum pH of the reaction.

The temperature-dependent activities of BglucH and BglucLEH were studied over the temperature range of 20–80 °C. As shown in Fig. 5b, the optimum temperature for BglucH and BglucLEH was around at 40 °C. The enzyme activities of both BglucH and BglucLEH gradually decreased when the temperature was elevated above 40 °C. The BglucLEH was, however, found to retain more than 40% of its activity at 60 °C, while BglucH retained only 3%, suggesting that ELP tag may increase the thermal stability of BglucH.

### Kinetic parameters of BglucH and BglucLEH

The kinetic parameters of BglucH and BglucLEH were conducted using *p*-nitrophenyl-β-d-glucopyranoside (*p*-NPG) as substrate under optimal conditions (Table 2). A Lineweaver–Burk plot was used to calculate the *K*_m_ and *V*_max_ values (Additional file 1: Figure S4). The affinity (*K*_m_) of BglucLEH for *p*-NPG was 5.27 mM, which was slightly lower than that of BglucH (5.73 ± 0.4 mM). The catalytic efficiency (*K*_cat_/*K*_m_) of BglucLEH (14.79 ± 0.5 S^−1^ mM^−1^) for *p*-NPG was higher than that of BglucH (12.10 ± 0.7 S^−1^ mM^−1^). The *V*_m_ of the BglucH and BglucLEH for *p*-NPG were 0.037 and 0.098 mM min^−1^, respectively.

**Table 2:** Kinetic parameters of BglucH and BglucLEH in hydrolysis of *p*-NPG

Enzyme	*K*_m_ (mM)	*K*_*ca*t_ (S^−1^)	*V*_m_ (mM min^−1^)	*K*_cat_/*K*_m_ (S^−1^mM^−1^)
BglucH	5.73 ± 0.4	69.36 ± 1.1	0.037 ± 0.006	12.10 ± 0.7
BglucLEH	5.27 ± 0.5	77.94 ± 0.9	0.098 ± 0.003	14.79 ± 0.5

### ELP tag increased protein stability

**Fig. 6:**
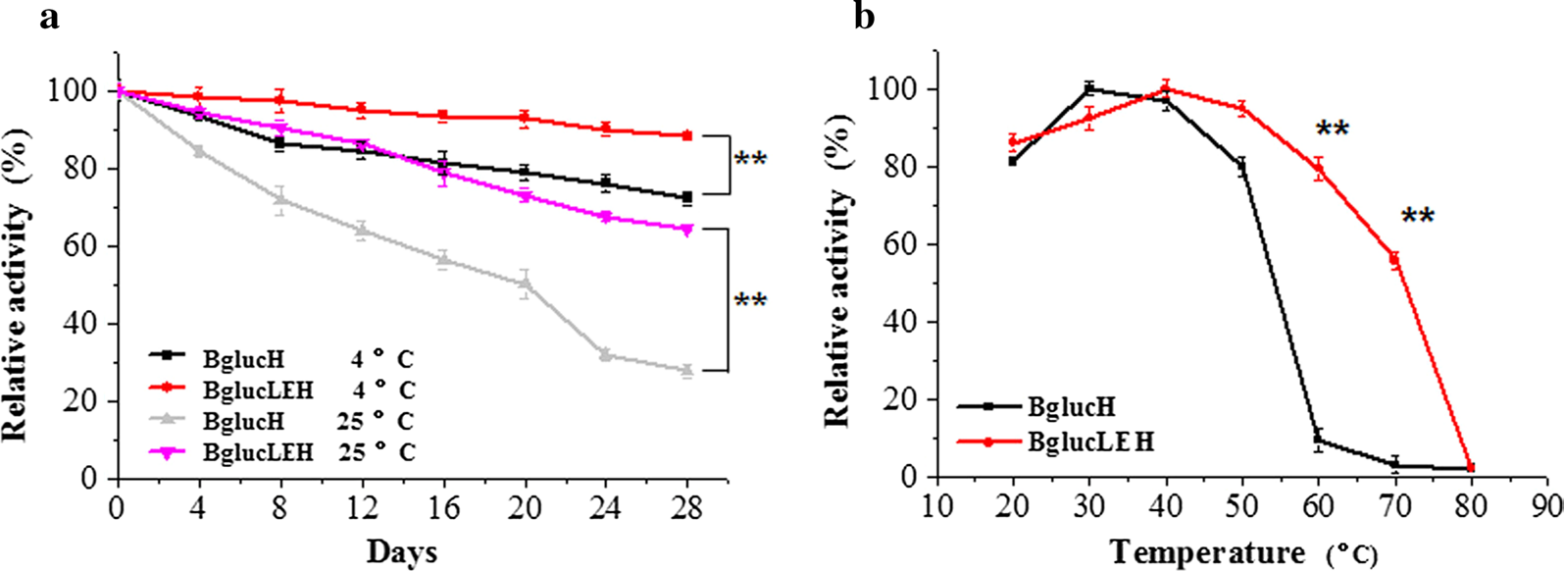
The experiment of stability. Storage stability dependent on time and temperature (**a**). Thermal stability (**b**). Each value in the panel represents the mean ± SD (*n* = 3). Asterisks denote statistical significance. *p < 0.05, **p < 0.01

The purified BglucH and BglucH EH were stored at 4 °C and 25 °C for 28 days. The relative enzyme activities were evaluated every 4 days as shown in Fig. 6a. The purified BglucLEH and BglucH retained 90% and 75% of its original activity when stored at 4 °C for 28 days, respectively. BglucH lost 70% of its enzyme activity at 25 °C at the 28th day, while BglucLEH lost only about 30% of its activity.

To study the thermal stability, the BglucH and BglucLEH were incubated at various temperatures for 30 min. As shown in Fig. 6b, BglucH and BglucLEH lost 90% and 20% of its initial activity when the temperature reached 60 °C, respectively. When the temperature reached 70 °C, BglucH lost almost all activity, while BglucLEH retained 55% of its initial activity. The results indicated that the thermal stability and storage stability of BglucLEH were higher than that of BglucH. Moreover, the effect of pH on the stability of BglucH and BglucLEH was measured on enzymes after incubation in buffer of different pH at 40 °C for 30 min. It was, however, observed that the pH stability of BglucLEH was not different from that of BglucH (data not shown), which indicated that ELP did not improve the enzyme’s pH tolerance.

**Fig. 7:**
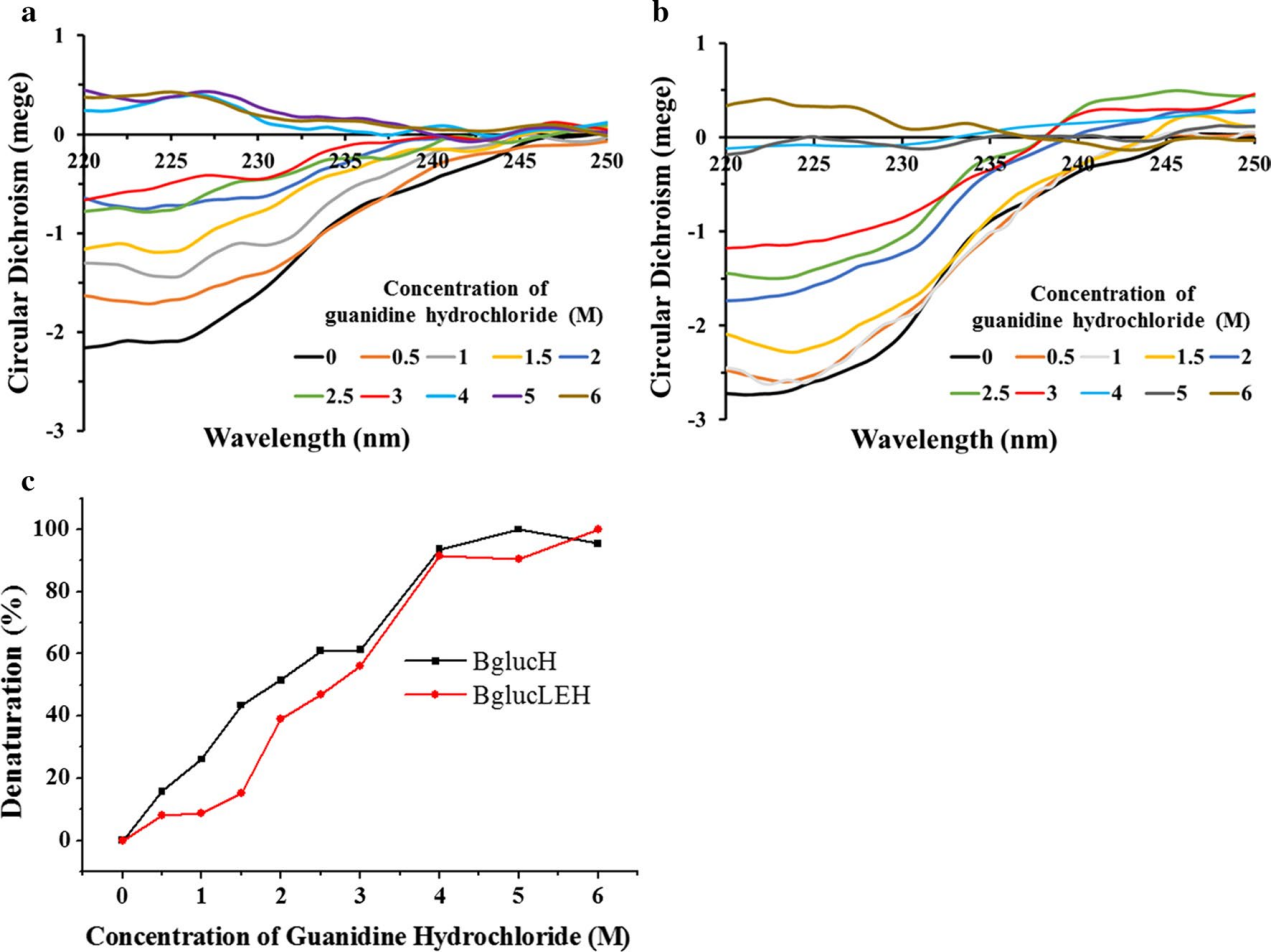
The circular dichroism spectra of BglucH (**a**) and BglucLEH (**b**) treated with various concentration of guanidine hydrochloride. The denaturation curve of BglucH and BglucLEH at 230 nm treated with various concentration of guanidine hydrochloride (**c**)

Guanidine hydrochloride is a common protein denaturant. In this study, BglucH and BglucLEH were incubated with various concentrations of guanidine hydrochloride, and then the changes in secondary structure were monitored. The Fig. 7a, b shows the CD spectra of BglucH and BglucLEH treated with various concentrations of guanidine hydrochloride. The denaturation curve (Fig. 7c) indicates that BglucH lost 15.7%, 26.1%, 43.4% of its secondary structure at 0.5, 1, 1.5 M guanidine hydrochloride, while the secondary structure of BglucLEH lost only 7.9%, 8.5%, 15.0% at 0.5, 1, 1.5 M guanidine hydrochloride, respectively. Taken together, ELP tag increased the protein stability against both heat and chaotropic denaturant.

## Discussion

Wood-feeding termites are well known as efficient cellulose decomposers that have the ability to degrade lignocelluloses with their unique enzyme complexes. Bgluc is known as a multifunctional enzyme for social maintenance in terms of both cellulose digestion and social communication in termites [19]. Bgluc from termites, such as *C. formosanus* [20, 21], *C. gestroi* [22, 23], *C. secundus* [24], *R. flavipes* [25], *M. barneyi* [26], *N. koshunensis* [27], *N. takasagoensis* [28, 29] have been identified. All of the reported Bglucs from termites were identified as GH1 [30]. In our previous study, three types of Bgluc homologs (CfGlu1C, CfGlu1B, CfGlu1D) from the termite *C. formosanus* were identified, and CfGlu1B has a high degree of amino acid similarity with the Bgluc from *O. formosanus*, *N. koshunensis*, *C. gestroi*, and *T. reesei* [5]. Moreover, Bgluc form *R. speratus* (RsBGI) has also been identified to be 86% identical to Bgluc from *C. formosanus* CfGlu1B [19].

Bgluc from some other organisms has been heterologously expressed and purified. The purity of Bgluc from *N. takasagoensis* (G1mgNtBG1) using Ni-NTA resin increased by 6.7-fold with a recovery rate of 83%, and a specific activity of 5.83 U/mg [29]. The yield and purification fold of Bgluc from *M. annandalei* using Ni-NTA resin were found to be 7.5% and 19.1-fold, respectively [30]. The yield and purification fold of Bgluc from Chinese snail using Ni-NTA resin were 21.0% and 9.0-fold, respectively [31]. The specific activity and yield of Bgluc from *T. amestolkiae* using HiTrap Capto Adhere, Mono Q 5/50 and Superdex 75 HR 10/30 were 50.4 U/mg, 78.5 U/mg, 82.6 U/mg, and 76.5%, 57.3%, 6.3%, respectively [32]. The purification fold and yield of the Bgluc from *M. thermophila* using gel filtration on Sephacryl S-100 were 11.2-fold and 25.5%, respectively [33]. The Bgluc from *P. piceum* was 8.7-fold with a specific activity of 80 U/mg against *p*-NPG using gel filtration chromatography [34]. In this study, our data showed that the specific activity, yield and purification fold of purified BglucLEH by ITC and Ni-NTA resin were 75.50 U/mg, 77.78%, 12.60-fold; and 68.22 U/mg, 79.07%, 11.39-fold, respectively. The results indicate that the purification performance of ITC was equivalent to or better than that of Ni-NTA resin. Performance of several rounds of ITC indicated that a heterogeneous solution of proteins gradually become more homogeneous, eventually resulting in substantially higher purity of recombinant ELP fusion protein [35]. However, our data showed that the purity of BglucLEH by two rounds of ITC did not significantly increase compared with a single round of ITC. It, however, reduced the yield of ELP fusion protein. One round of ITC was sufficient for BglucLEH purification. In summary, the purification performance of ITC had a comparative advantage, that is, been simple, time efficient, inexpensive and highly efficient, compared with conventional column chromatography. A comparison of the biochemical properties and kinetic performance of Bgluc from different species to *p*-NPG is shown in Table 3. Bgluc from fungi tends to have high catalytic performance compared to that of other species. Commercial cellulases are mainly from fungi, especially *T. reesei*. Bgluc produced by other species must be added to a *T. reesei* enzyme mixture to increase the efficiency of the hydrolysis of cellulosic substrates [36]. The catalytic efficiency of Bgluc from termites (such as *R. flavipes* [25], *C. gestroi* [22], *M. annandalei* [30] and *C. formosanus* [5]) tends to be lower compared to Bgluc from fungi [32, 37–43]. In termites, it was reported that the optimum pH and temperature of Bgluc were within pH 5.0–6.5 and 40–60 °C, respectively [5, 22, 25–27, 30]. Here, the optimum pH and temperature of BglucH and BglucLEH were identified as 5.5 and 40 °C.

**Table 3:** Enzymatic properties of Bgluc from termites and other species to *p*-NPG

Source organism	Optimum temperature (°C)	Optimum pH	*K*_m_ (mM)	*K*_cat_ (s^−1^)	*K*_cat_/*K*_m_ (s^−1^ mM^−1^)	References
*Talaromyces leycettanus*	75	4.5	0.18	1664	9096	[37]
*Trichoderma reesei*	40	5	0.09	118	1311	[38]
*Talaromyces amestolkiae*	40	5	0.41	485	1167	[32]
*Aspergillus japonicus*	40	5	0.6	259	431.7	[38]
*Aspergillus niger*	40	4	2.2	917	416.8	[39]
*Myceliophthora thermophila*	40	5	0.39	147	377	[40]
*Thermoascus aurantiacus*	60	4.5	0.11	242	2200	[41]
*Penicillium verruculosum*	40	5	0.44	160	363.6	[42]
*Fomitopsis palustris*	50	5	0.12	721	6008	[43]
*Nasutitermes takasagoensis*	65	5.5	0.67	ND	ND	[29]
*Reticulitermes flavipes*	40	7	1.66	22.92	13.80	[25]
*Neotermes koshunensis*	40	5	0.77	ND	ND	[27]
*Coptotermes gestroi*	55	5	1.59	57.19	35.99	[22]
*Macrotermes barneyi*	45	5	ND	ND	ND	[26]
*Microcerotermes annandalei*	50–55	5–6	1.47	8.98	6.12	[30]
*Globitermes sulphureus*	90	6	0.18	ND	ND	[44]
*Coptotermes formosanus*	40	5	2.20	60.7	27.0	[5]
*Coptotermes formosanus*	40	5.5	5.73	69.36	12.10	This work

*ND* not determined

CD analysis of Bgluc from *C. gestroi* (CgBG1) showed a negative peak (α-helical) at 222 nm and a positive peak (β-sheets) at ~ 192 nm. Based on the CgEG1 model, the secondary structure of Bgluc was primarily α-helical (43.6%) followed by β-sheets (13.6%) and unstructured loops (42.8%) [22]. Our results showed one positive peak at 196 nm (β-sheets) and two negative peaks (α-helical) at 208 nm and 222 nm. The CD analysis of BGL-2 (Bgluc from *T. amestolkiae*) revealed that all BGL-2 forms have a typical spectrum of *α* + *β* folded structures, and the spectrum of BGL-2T* (a mutation of BGL-2) lacked the minimum at 208 nm, a typical signature of proteins with cellulose binding domain, which may be one of the reasons for its increased stability to temperature and pH [32].

ELP fusion proteins can increase proteins’ solubility and maintain its biological activity as well as its physical properties [45–48]. Floss et al. [49] reported that scFv ELP fusions can accumulate to very high levels, but the ELP does not affect the protein assembly, folding or the antigen-binding properties of antibodies. The antibody–ELP fusion proteins have been reported to retain the parent antibody’s ability to bind antigen, albeit with slightly reduced potency [45]. A levansucrase–ELP fusion protein was expressed in *E. coli*, and levansucrase activity was not interfered by the ELPs tag [47]. The fusion protein comprising of ELP and KGF retained the functional characteristics of KGF and elastin as evidenced by its enhancement of keratinocyte and fibroblast proliferation [50]. Here, our data showed that ELP did not affect the protein production, enzyme activity or the kinetic parameters of BglucH.

The guest residue composition and chain length of ELP are two parameters that affect the T_*t*_. The hydrophobic guest residues and more chain lengths is associated with lower *T*_t_, while the hydrophilic guest residues and less chain lengths produce higher *T*_t_. Furthermore, placing the ELP at the C-terminus (protein–ELP) or N-terminus (ELP–Protein) of the target protein controls expression level and activity of elastin-like polypeptide fusion proteins [51]. Protein–ELP fusions have a greater level of expression and specific activity levels than ELP–protein fusions [51, 52]. One explanation is that ELP–protein increases the fraction of misfolded, and less active conformers, which are also preferentially degraded compared to C-terminus ELP fusion (protein–ELP) [51]. Here, we designed a 50 repeating pentapeptide sequence, Val–Pro–Gly–Val–Gly (VPGVG)_50_, and placed the ELP at the C-terminus of Bgluc.

Bgluc has attracted the attention of researchers to improve the enzyme’s activity and thermostability through bioengineering technology due to its multifunctional role and industrial usefulness. For example, Xia et al. [37] identified and overexpressed a novel Bgluc with high specific activity and high catalytic efficiency in *P. pastoris*, and enhanced the pH stability by reducing the *O*-glycosylation. Liu et al. [53] demonstrated a combinatorial selection/screening strategy for finding thermostable Bgluc mutants. The Bgluc mutations from *T. reesei* resulted in shift of optimal temperature to 50 °C, at which the wild type was almost inactive [54]. Moreover, the Bgluc mutant VM2 from *T. fusca* with three amino acid changes has been reported to be more thermostable [55].

ELP fusion proteins have enhanced thermal stability and improved the circulating half-life and long-term efficacy of therapeutic ELP fusion proteins [56]. To improve the stability of engineered biopolymers against denaturing agents and oxidants, the biopolymers can be fused to enzymes to protect them from decomposition, while maintaining their molecular integrity over an extended period of time. A study by Liu et al. [57] fused collagen-like polypeptide (CLP) to an ELP, and separately fused them to the enzymes SOD and DAAO. The result showed that SOD–CLP–ELP and DAAO–CLP–ELP were more resistant to urea denaturation than SOD and DAAO alone. Udo et al.’s team showed that therapeutic proteins fused with ELP possessed a substantially longer serum half-life compared to the non-ELP fusion proteins [58]. ELP forms hydrogen bonds not only with water molecules, but also with other ELP molecules [59]. The longer the ELP sequence repeats, the more hydrogen bonds it contains which provide the most general explanation for the thermal stability in proteins [60]. Cho et al.’s team also speculated from their data that hydrogen bonding related to structure formation rather than hydrophobicity and was the key factor in the stabilization of collapsed state of ELPs [59]. Furthermore, the organized reversible particles formed by ELP at high temperature will restrict the movement of the enzyme covalently linked, to prevent the enzyme form irreversible aggregation at high temperature. We also demonstrated that the ELP fusion enzyme has higher stability to denaturants compared to the enzyme without ELP. The denaturant competes to form hydrogen bond with amino acids in protein instead of the protein internal hydrogen bonds which stabilizes protein.

ELP-mediated purification may prove as an inexpensive and highly efficient form of purification, but further removal of ELP tags remains a challenge. To solve this problem, the Chilkoti lab [61], Wood lab [62, 63] and Chen lab [64] combined ELP proteins with self-cleaving inteins, which significantly simplified the process. From this study, however, we found that ELP tag neither affected the biological activity of the enzyme nor its structural integrity but rather improved its stability, and as a result removal of the ELP tag from the purified enzyme was not of prime importance for this protein.

We also examined the reusability of the BglucLEH, and the result showed that the purification capacity of the BglucLEH was below 20% in second purification cycles compared with first purification cycles (data not shown), which indicated that ELP did not increase the enzyme’s reusability. Immobilization of enzyme is an applied technique that improves the stability and reusability of an enzyme, making it an economically feasible process [65, 66]. Immobilization using a covalent coupling is found to be better than a physical adsorption in binding strength. In our previous study, a novel Fe_3_O_4_/PMG/IDA-Ni^2+^ nanoparticles were synthesized, and protein with His tag was immobilized onto their surface through combination with the imidazole group and Ni^2+^ [65]. The binary ELP&His tag could also be used to immobilize BglucLEH onto Fe_3_O_4_/PMG/IDA-Ni^2+^ nanoparticles in addition to purification and thereby increasing its stability. The excellent reusability of immobilized enzymes is of great importance as it decreases the cost of industrial applications.

Taking into account their substrate specificity, Bgluc has been classified into three groups: cellobiases (high specificity for cellobiose), aryl-β-glucosidases (high specificity for substrates such as *p*-NPG), or wide range Bgluc, which exhibit broad activities on various types of substrates [67]. Bgluc from *C. formosanus* shows higher affinity and activity toward cellobiose and cellotriose than other substrates [20]. The G1mgNtBG1 has broad substrate specificity and hydrolyzes both natural and synthetic substrates [29]. The Bgluc form *G. sulphureus* was active on the *p*-NPG and cellobiose, but inactive on avicel [44]. Bgluc from *R. flavipes* was most active against *p*-NPG, cellobiose, salicin, and laminaribose [25]. Our previous study revealed that BglucH showed high activity toward *p*-NPG, cellobiose, cellotriose and lactosum [5]. Here, the Bgluc activity was measured using *p*-NPG, because its hydrolysis can be directly quantified in colorimetric assays. It should be noted that there are several limitations because the activity of Bgluc was only examined on *p*-NPG, and it would be interesting to have an activity with cellobiose or cellotriose that are the natural substrate of Bgluc. To improve the large-scale application of the biocatalyst in biomass conversion, lignocellulose saccharification experiments is necessitated in further research.

## Conclusions

Bgluc, an important member of the cellulose system, plays a critical role in the enzymatic hydrolysis of biomass for the production of biofuels. In this study, a novel binary ELP&His tag was fused with Bgluc from *C. formosanus* to construct a Bgluc–linker–ELP–His recombinant fusion protein (BglucLEH). The recovery rate and purification fold of the fusion enzyme BglucLEH reached 77.78% and 12.60, respectively, through ITC with 0.5 M (NH_4_)_2_SO_4_ based on the ELP tag. The purification by ITC was superior to the traditional Ni-NTA resin. Moreover, the ELP did not affect the enzyme activity, kinetic parameters and secondary structure of Bgluc. More importantly, ELP improved the Bgluc stability in harsh conditions such as heating and exposure to denaturant.

## Materials and methods

### Materials

The expression vector pET28a(+) and *E. coli* strains BL21 (DE3), were stored at − 80 °C in our lab. Kanamycin, protease inhibitors phenylmethanesulfonyl fluoride (PMSF) and IPTG were purchased from Sangon Biotech (Shanghai, China). *p*-NPG was purchased from Bio Teke Corporation (Beijing, China). Glycine, Tris, SDS, Bromophenol blue, coomassie brilliant blue R 250 and *p*-nitrophenol (*p*-NP) were purchased from San gon Biotech (Shanghai, China). Acrylamide and TEMED were bought from Sigma (USA). Glycerinum, isopropanol, methyl alcohol, β-mercaptoethanol, ammonium persulfate and glacial acetic acid were obtained from Sinopharm Chemical Reagent (Shanghai, China). Thermo Scientific Pierce BCA Protein Assay Kit was purchased from Thermo Fisher Scientific (MA, USA). High Affinity Ni-NTA Resin was bought from Genscript (Nanjing, China). All other reagents were of analytical grade and used without any further treatment.

### Construction of recombinant expression plasmids (BglucLEH and BglucH)

The amino acid coding sequences for Bgluc of *C. formosanus* was derived from gene (GQ911585) at GenBank NCBI. The 50 repeating pentapeptide sequence, Val–Pro–Gly–Val–Gly (VPGVG)_50_, was designed (denoted as ELP). The Bgluc and ELP genes were fused together with a flexible linker (GGGGS)_3_ and adding a 6xHis-tag to the 3′ of the fusion gene. The schematic diagram, nucleotide sequence and restriction enzyme sites are shown in Fig. 1. The nucleotide sequence of the designed fusion protein was synthesized by Synbio Tech (Jiangsu, China), and subcloned into pET28a(+) resulting in the recombinant expression of the plasmid pET28a(+)-BglucLEH (Bgluc–Linker–ELP–His) (Fig. 1). The recombinant plasmid pET28a(+)-Bgluc (BglucH) expressing a His-tagged Bgluc was previously constructed in our lab [5].

### Production of recombinant proteins (BglucLEH and BglucH)

The constructed recombinant expressional plasmids pET28a(+)-BglucLEH, pET28a(+)-BglucH and empty vector pET28a(+) were transformed into the *E. coli* strain BL21 (DE3) for protein production. A freshly transformed single colony was inoculated in LB media with 50 μg/mL kanamycin and cultured overnight in an orbital shaker at 37 °C with shaking (200 rpm). Two milliliters of this culture was inoculated into 200 mL of fresh medium supplemented with 50 μg/mL kanamycin. When OD_600_ of culture reached 0.4, IPTG was then added to a final concentration of 1 mM, and the cells were grown at 25 °C or 37 °C for 16–20 h. Cells were harvested by centrifuging at 4500×*g* at 4 °C for 20 min, and cell pellets were stored at − 80 °C. The cell pellets were thawed and resuspended in 10 mL Tris–HCl (50 mM, pH 8.0) and the cells were washed twice with Tris–HCl. Finally, the cells were resuspended in 5 mL Tris–HCl buffer with 1 mM PMSF. The cells were broken by sonication for 30 min using ultrasonic cell disruptor, with alternately sonication at 10 s and intermittent periods of cooling 10 s. The supernatant and precipitate were separated by centrifugation twice at 12,000×*g* for 20 min at 4 °C.

### Purification of fusion proteins

#### (1) Purification by Ni-charged resin (BglucLEH and BglucH)

The recombinant proteins (BglucLEH and BglucH) were purified with Ni-NTA resin because both the BglucLEH and BglucH contain His tag. Purification conditions followed the manufacturer’s instructions. Briefly, the crude cell lysates were mixed with the Ni-NTA resin and incubated at 4 °C for 2 h. Subsequently, the surplus cell lysates flowed through the column while target protein was retained. Ni-NTA resin was washed with 3-mL solution I (50 mM Tris, pH 8.0, 300 mM NaCl, 50 mM imidazole), then 3-mL solution II (50 mM Tris, pH8.0, 300 mM NaCl, 100 mM imidazole). Finally, His-tagged protein (BglucLEH or BglucH) was eluted from Ni-charged resin with 2-mL solution III (50 mM Tris, pH 8.0, 300 mM NaCl, 400 mM imidazole). The eluted protein was dialyzed against dialysis buffer (50 mM Tris–HCl, pH 8.0) at 4 °C to remove imidazole. The concentration of purified proteins (BglucLEH and BglucH) was determined by the Pierce^®^ BCA Protein Assay Kit.

#### (2) Purification by ITC (BglucLEH)

BglucLEH also was purified with different salt (the salt being investigated at its specified concentration, dissolved in water) including 1 M NaH_2_PO_4_, 1 M (NH_4_)_2_SO_4_, 1 M Na_2_SO_4_, 2.5 M NaCl because BglucLEH contains ELP tag. After SDS–PAGE analysis, (NH_4_)_2_SO_4_ was selected as the purification salt.

To determine the concentration of (NH_4_)_2_SO_4_, BglucLEH was purified with various concentrations of (NH_4_)_2_SO_4_ (0.3, 0.5, 0.7 and 1.0 M). First, a certain amount of (NH_4_)_2_SO_4_ was added into 500-μL crude extract, and the mixed solution was incubated for 20 min and then centrifuged at 12,000×*g* for 10 min at room temperature (25 °C) (termed ‘‘hot spin’’). The supernatant was withdrawn and discarded, and the pellet containing BglucLEH was redissolved in cold Tris–HCl buffer at 4 °C for 30–60 min. Then, a centrifugation step at 4 °C for 10 min (termed ‘‘cold spin’’) is carried out to remove contaminants that may have been physically trapped in BglucLEH. The supernatant containing BglucLEH supernatant from the cold spin is retained, and the pellet is discarded. This process constitutes one round of ITC. The concentration of protein (BglucLEH) was determined by the Pierce^®^ BCA Protein Assay Kit. The recovery rate and purification refold of fusion proteins were determined by enzyme activity measurement.

$$\begin{aligned} {\text{Recovery rate (\%) }}&=\frac{{{\text{Purified enzyme activity}}}}{{{\text{Total enzyme activity in crude extract}}}} \times 100 \hfill \\ {\text{Purification fold}} &= \frac{{{\text{Specific activity of purified enzyme}}}}{{{\text{Specific activity of crude enzyme}}}} \hfill \\ \end{aligned}$$

### Enzyme activity assay

The activity of Bgluc was measured as described in our previous study [65]. Briefly, a certain amount of BglucLEH or BglucH was incubated with 5 mM *p*-NPG, 50 mM NaAc–HAc (pH 5.5) buffer at 40 °C. Assays were terminated 10 min later by the addition of 1 M Na_2_CO_3_. The amount of *p*-NP released was determined at 410 nm with spectrophotometer. The specific activity (U) was defined as the amount of enzyme that releases 1 μmol of catalytic product *p*-NP per min.

### Kinetic parameter activity assay

The kinetic parameters such as the Michaelis constant (*K*_m_), maximum velocity (*V*_max_) and turnover number (*k*_cat_) for BglucH and BglucLEH enzymes were measured using *p*-NPG at 1–14 mM. Reactions were carried out in 50 mM sodium acetate buffer (pH 5.5) with purified BglucH and BglucLEH at 40 °C and were stopped by adding 1 M Na_2_CO_3_. The *K*_m_ and *V*_max_ were calculated according to the Lineweaver–Burk method.

### Secondary structure of BglucH and BglucLEH

The CD spectra of BglucLEH enzyme and BglucH were recorded at a wavelength of 190–250 nm with a JASCO-810 spectropolarimeter (Jasco, Tokyo, Japan) and a quartz cell with 0.1 mm optical path length. All spectra were corrected by subtracting a blank spectrum (buffer without enzyme). The purified enzyme BglucH and BglucLEH solutions were prepared by dissolving the enzymes in PBS buffer to a final concentration of 0.1 mg/mL. The measurements were performed at 25 °C.

### Effect of pH and temperature on BglucH and BglucLEH Activity

*p*-NPG was used as the substrate for activity assays to investigate optimum temperature and pH. The purified BglucH and BglucLEH were incubated with substrate (*p*-NPG) at various temperatures that ranged from 20 to 80 °C (pH 5.5) or in buffers over a pH range of 3.0–8.0 (40 °C) for 10 min. Then, the catalytic activity was then measured as described above.

### Enzyme stability

The stability of BglucH and BglucLEH was investigated, which included storage, thermal stability and resistance to chemical denaturant. For storage stability, purified BglucH and BglucLEH were stored in 4 °C and 25 °C, and the specific enzyme activity was measured at 4 °C and 25 °C every 4 days for 28 days. The residual specific enzyme activity of BglucH and BglucLEH reduced over time in comparison to enzyme activity measured initially. The results presented are the average of three independent experiments.

The thermal and pH stability of BglucH and BglucLEH were measured according to the method described in our previous studies [65, 66]. Briefly, the purified BglucH and BglucLEH were pre-incubated without substrate (*p*-NPG) at various temperatures (20, 30, 40, 50, 60, 70, 80 °C) and pH 5.5 for 30 min. Then, the residual enzyme activities of BglucH and BglucLEH were evaluated using *p*-NPG at under standard conditions (pH 5.5, 40 °C and 10 min). To estimate pH stability, the enzyme was pre-incubated in buffers over a pH range of 3.0–8.0 without substrate at 40 °C for 30 min, and the residual activities were measured using *p*-NPG under the standard conditions (pH 5.5, 40 °C and 10 min). The maximum activity value was set as value obtained by enzymes exposed to optimum temperature.

To assess the BglucH and BglucLEH resistance to chemical denaturant, the purified BglucH and BglucLEH were incubated with various concentrations of chaotropic reagent guanidine hydrochloride (0, 0.5, 1, 1.5, 2, 2.5, 3, 4, 5, 6 M) at room temperature for 30 min, respectively. The CD analysis was then measured as described above.

## Additional file

**Additional file 1: Figure S1.** SDS-PAGE analysis of BglucH and BglucLEH enzyme. (a): SDS–PAGE analysis of BglucH enzyme and BglucLEH enzyme induce at 37 °C. Lane M, Protein molecular weight marker (Broad); Lane 1, cell lysates of BglucH induced 0 h; Lane 2, cell lysates of BglucH induced 8 h; Lane 3, precipitation lysates containing BglucH; Lane 4, crude cell lysate of BglucH; Lane 5, crude cell lysate of empty pET-28a(+) vector; Lane 6, cell lysates of BglucLEH induced 0 h; Lane 7, cell lysates of BglucLEH induced 8 h; Lane 8: precipitation lysates containing BglucLEH; Lane 9, crude cell lysate of BglucH; The arrows in the figure are the insoluble fractions of the BglucH enzyme and the BglucLEH enzyme. (b): SDS–PAGE analysis of BglucH enzyme and BglucLEH enzyme induced at 25 °C. Lane M, Protein molecular weight marker (Broad); Lane 1, cell lysates of BglucH induced 0 h; Lane 2, cell lysates of BglucH induced 8 h; Lane 3, precipitation lysates containing BglucH; Lane 4, crude cell lysate of BglucH; Lane 5, crude cell lysate of empty pET-28a(+) vector; Lane 6, cell lysates of BglucLEH induced 0 h; Lane 7, cell lysates of BglucLEH induced 8 h; Lane 8: precipitation lysates containing BglucLEH; Lane 9, crude cell lysate of BglucH; The red wireframe in the figure indicates the whole bacteria, sediment and supernatant solution containing BglucH enzyme and BglucLEH enzyme, respectively. **Figure S2.** SDS–PAGE analysis of BglucH induced at 25 °C and purification by Ni-NTA resin. Lane M, Protein molecular weight marker (Broad); Lane 1, crude cell lysate of cell with empty pET-28a(+) vector; Lane 2, crude cell lysate containing BglucH; Lanes 3–4, purified BglucH by Ni-NTA resin. **Figure S3.** SDS–PAGE analysis of twice ITC and 400mM imidazole purified BglucLEH. Lane M, Protein molecular weight marker (Broad); Lane 1, crude cell lysate of cell with empty pET-28a(+) vector; Lane 2, crude cell lysate containing BglucLEH; Lanes 3–4, BglucLEH purified after one and two rounds of ITC operation using 0.5 M of (NH_4_)_2_SO_4._ **Figure S4.** Lineweaver–Burk plots for (a) BglucLEH, (b) BglucLEH.

## Data Availability

β-Glucosidase sequence is deposited in GenBank under the Accession Number GQ911585.

## Abbreviations

Bgluc: β-glucosidase
BglucH: Bgluc–His
ELP: elastin-like polypeptide
BglucLEH: Glu–linker–ELP–His
ITC: inverse transition cycling
T_*t*_: phase transition temperature
CD: circular dichroism
*p*-NPG: *p*-nitrophenyl-β-d-glucopyranoside
CLP: collagen-like polypeptide
PMSF: phenylmethanesulfonyl fluoride
IPTG: isopropyl-beta-d-thiogalactopyranoside
*p*-NP: *p*-nitrophenol

## References

[1] Ni JF, Tokuda G (2013). Lignocellulose-degrading enzymes from termites and their symbiotic microbiota. Biotechnol Adv.

[2] Ferreira RDG, Azzoni AR, Freitas S (2018). Techno-economic analysis of the industrial production of a low-cost enzyme using *E. coli*: the case of recombinant beta-glucosidase. Biotechnol Biofuels.

[3] Kuusk S, Valjamae P (2017). When substrate inhibits and inhibitor activates: implications of beta-glucosidases. Biotechnol Biofuels.

[4] Tokuda G, Watanabe H, Hojo M, Fujita A, Makiya H, Miyagi M (2012). Cellulolytic environment in the midgut of the wood-feeding higher termite *Nasutitermes takasagoensis*. J Insect Physiol.

[5] Feng T, Liu H, Xu Q, Sun J, Shi H (2015). Identification and characterization of two endogenous beta-glucosidases from the termite *coptotermes formosanus*. Appl Biochem Biotechnol.

[6] Liew KJ, Lim L, Woo HY, Chan KG, Shamsir MS, Goh KM (2018). Purification and characterization of a novel GH1 beta-glucosidase from *Jeotgalibacillus malaysiensis*. Int J Biol Macromol.

[7] Mendez-Liter JA, de Eugenio LI, Prieto A, Martinez MJ (2018). The beta-glucosidase secreted by *Talaromyces amestolkiae* under carbon starvation: a versatile catalyst for biofuel production from plant and algal biomass. Biotechnol Biofuels.

[8] Gao J, Qian Y, Wang Y, Qu Y, Zhong Y (2017). Production of the versatile cellulase for cellulose bioconversion and cellulase inducer synthesis by genetic improvement of *Trichoderma reesei*. Biotechnol Biofuels.

[9] Tiwari R, Singh PK, Singh S, Nain PKS, Nain L, Shukla P (2017). Bioprospecting of novel thermostable beta-glucosidase from *Bacillus subtilis* RA10 and its application in biomass hydrolysis. Biotechnol Biofuels.

[10] Yeboah A, Cohen RI, Rabolli C, Yarmush ML, Berthiaume F (2016). Elastin-like polypeptides: a strategic fusion partner for biologics. Biotechnol Bioeng.

[11] Dan EM, Chilkoti A (1999). Purification of recombinant proteins by fusion with thermally-responsive polypeptides. Nat Biotechnol.

[12] Christensen T, Trabbic-Carlson K, Liu W, Chilkoti A (2007). Purification of recombinant proteins from *Escherichia coli* at low expression levels by inverse transition cycling. Anal Biochem.

[13] Araújo A, Olsen BD, Machado AV (2018). Engineering elastin-like polypeptide-poly(ethylene glycol) multiblock physical networks. Biomacromol.

[14] Christensen T, Hassouneh W, Trabbic-Carlson K, Chilkoti A (2013). Predicting transition temperatures of elastin-like polypeptide fusion proteins. Biomacromol.

[15] Gutiérrez SP, Saberianfar R, Kohalmi SE, Menassa R (2013). Protein body formation in stable transgenic tobacco expressing elastin-like polypeptide and hydrophobin fusion proteins. BMC Biotechnol.

[16] Shi C, Meng Q, Wood DW (2013). A dual ELP-tagged split intein system for non-chromatographic recombinant protein purification. Appl Microbiol Biotechnol.

[17] Lin F, Yan D, Chen Y, E FE, Shi H, Han B (2018). Cloning, purification and enzymatic characterization of recombinant human superoxide dismutase 1 (hSOD1) expressed in *Escherichia coli*. Acta Biochim Pol.

[18] Huo J, Shi H, Yao Q, Chen H, Wang L, Chen K (2010). Cloning and purification of recombinant silkworm dihydrolipoamide dehydrogenase expressed in *Escherichia coli*. Protein Expr Purif.

[19] Shimada K, Maekawa K (2014). Gene expression and molecular phylogenetic analyses of beta-glucosidase in the termite *Reticulitermes speratus* (Isoptera: Rhinotermitidae). J Insect Physiol.

[20] Zhang D, Allen AB, Lax AR (2012). Functional analyses of the digestive beta-glucosidase of *Formosan subterranean* termites (*Coptotermes formosanus*). J Insect Physiol.

[21] Zhang DH, Lax AR, Bland JM, Yu JJ, Fedorova N, Nierman WC (2010). Hydrolysis of filter-paper cellulose to glucose by two recombinant endogenous glycosyl hydrolases of *Coptotermes formosanus*. Insect Sci.

[22] Cairo JP, Oliveira LC, Uchima CA, Alvarez TM, Citadini AP, Cota J (2013). Deciphering the synergism of endogenous glycoside hydrolase families 1 and 9 from *Coptotermes gestroi*. Insect Biochem Mol Biol.

[23] Franco Cairo JP, Leonardo FC, Alvarez TM, Ribeiro DA, Buchli F, Costa-Leonardo AM (2011). Functional characterization and target discovery of glycoside hydrolases from the digestome of the lower termite *Coptotermes gestroi*. Biotechnol Biofuels.

[24] Matsuura K, Yashiro T, Shimizu K, Tatsumi S, Tamura T (2009). Cuckoo fungus mimics termite eggs by producing the cellulose-digesting enzyme beta-glucosidase. Curr Biol.

[25] Scharf ME, Kovaleva ES, Jadhao S, Campbell JH, Buchman GW, Boucias DG (2010). Functional and translational analyses of a beta-glucosidase gene (glycosyl hydrolase family 1) isolated from the gut of the lower termite *Reticulitermes flavipes*. Insect Biochem Mol Biol.

[26] Wu Y, Chi S, Yun C, Shen Y, Tokuda G, Ni J (2012). Molecular cloning and characterization of an endogenous digestive beta-glucosidase from the midgut of the fungus-growing termite *Macrotermes barneyi*. Insect Mol Biol.

[27] Uchima CA, Tokuda G, Watanabe H, Kitamoto K, Arioka M (2011). Heterologous expression and characterization of a glucose-stimulated beta-glucosidase from the termite *Neotermes koshunensis* in *Aspergillus oryzae*. Appl Microbiol Biotechnol.

[28] Tokuda G, Miyagi M, Makiya H, Watanabe H, Arakawa G (2009). Digestive beta-glucosidases from the wood-feeding higher termite, *Nasutitermes takasagoensis*: intestinal distribution, molecular characterization, and alteration in sites of expression. Insect Biochem Mol Biol.

[29] Uchima CA, Tokuda G, Watanabe H, Kitamoto K, Arioka M (2012). Heterologous expression in *Pichia pastoris* and characterization of an endogenous thermostable and high-glucose-tolerant beta-glucosidase from the termite *Nasutitermes takasagoensis*. Appl Environ Microbiol.

[30] Arthornthurasuk S, Jenkhetkan W, Suwan E, Chokchaichamnankit D, Srisomsap C, Wattana-Amorn P (2018). Molecular characterization and potential synthetic applications of GH1 beta-glucosidase from higher termite *Microcerotermes annandalei*. Appl Biochem Biotechnol.

[31] Zouhar J, Nanak E, Brzobohaty B (1999). Expression, single-step purification, and matrix-assisted refolding of a maize cytokinin glucoside-specific beta-glucosidase. Protein Expr Purif.

[32] Mendez-Liter JA, Gil-Munoz J, Nieto-Dominguez M, Barriuso J, de Eugenio LI, Martinez MJ (2017). A novel, highly efficient beta-glucosidase with a cellulose-binding domain: characterization and properties of native and recombinant proteins. Biotechnol Biofuels.

[33] Bonfá EC, Maria DSMM, Gomes E, Bonilla-Rodriguez GO (2018). Biochemical characterization of an isolated 50 kDa beta-glucosidase from the thermophilic fungus *Myceliophthora thermophila* M.7.7. Biocatal Agric Biotechnol.

[34] Gao L, Gao F, Zhang D, Zhang C, Wu G, Chen S (2013). Purification and characterization of a new beta-glucosidase from *Penicillium piceum* and its application in enzymatic degradation of delignified corn stover. Bioresour Technol.

[35] McCarthy B, Yuan Y, Koria P (2016). Elastin-like-polypeptide based fusion proteins for osteogenic factor delivery in bone healing. Biotechnol Progr.

[36] Chen M, Zhao J, Xia LM (2008). Enzymatic hydrolysis of maize straw polysaccharides for the production of reducing sugars. Carbohydr Polym.

[37] Xia W, Xu X, Qian L, Shi P, Bai Y, Luo H (2016). Engineering a highly active thermophilic beta-glucosidase to enhance its pH stability and saccharification performance. Biotechnol Biofuels.

[38] Korotkova OG, Semenova MV, Morozova VV, Zorov IN, Sokolova LM, Bubnova TM (2009). Isolation and properties of fungal beta-glucosidases. Biochemistry (Mosc).

[39] Yan TR, Lin YH, Lin CL (1998). Purification and characterization of an extracellular beta-glucosidase II with high hydrolysis and transglucosylation activities from *Aspergillus niger*. J Agric Food Chem.

[40] Karnaouri A, Topakas E, Paschos T, Taouki I, Christakopoulos P (2013). Cloning, expression and characterization of an ethanol tolerant GH3 beta-glucosidase from *Myceliophthora thermophila*. PeerJ.

[41] Parry NJ, Beever DE, Owen E, Vandenberghe I, Van Beeumen J, Bhat MK (2001). Biochemical characterization and mechanism of action of a thermostable beta-glucosidase purified from *Thermoascus aurantiacus*. Biochem J.

[42] Krogh KB, Harris PV, Olsen CL, Johansen KS, Hojer-Pedersen J, Borjesson J (2010). Characterization and kinetic analysis of a thermostable GH3 beta-glucosidase from *Penicillium brasilianum*. Appl Microbiol Biotechnol.

[43] Yoon JJ, Kim KY, Cha CJ (2008). Purification and characterization of thermostable beta-glucosidase from the brown-rot basidiomycete *Fomitopsis palustris* grown on microcrystalline cellulose. J Microbiol.

[44] Wang Q, Qian C, Zhang XZ, Liu N, Yan X, Zhou Z (2012). Characterization of a novel thermostable beta-glucosidase from a metagenomic library of termite gut. Enzyme Microb Technol.

[45] Floss DM, Sack M, Arcalis E, Stadlmann J, Quendler H, Rademacher T (2009). Influence of elastin-like peptide fusions on the quantity and quality of a tobacco-derived human immunodeficiency virus-neutralizing antibody. Plant Biotechnol J.

[46] Meyer DE, Trabbic-Carlson K, Chilkoti A (2001). Protein purification by fusion with an environmentally responsive elastin-like polypeptide: effect of polypeptide length on the purification of thioredoxin. Biotechnol Progr.

[47] Kang HJ, Kim JH, Chang WJ, Kim ES, Koo YM (2007). Heterologous expression and optimized one-step separation of levansucrase via elastin-like polypeptides tagging system. J Microbiol Biotechnol.

[48] Chow DC, Dreher MR, Trabbic-Carlson K, Chilkoti A (2006). Ultra-high expression of a thermally responsive recombinant fusion protein in *E. coli*. Biotechnol Prog.

[49] Floss DM, Sack M, Stadlmann J, Rademacher T, Scheller J, Stoger E (2008). Biochemical and functional characterization of anti-HIV antibody-ELP fusion proteins from transgenic plants. Plant Biotechnol J.

[50] Koria P, Yagi H, Kitagawa Y, Megeed Z, Nahmias Y, Sheridan R (2011). Self-assembling elastin-like peptides growth factor chimeric nanoparticles for the treatment of chronic wounds. Proc Natl Acad Sci USA.

[51] Christensen T, Amiram M, Dagher S, Trabbic-Carlson K, Shamji MF, Setton LA (2009). Fusion order controls expression level and activity of elastin-like polypeptide fusion proteins. Protein Sci.

[52] Fletcher EE, Yan D, Kosiba AA, Zhou Y, Shi H (2019). Biotechnological applications of elastin-like polypeptides and the inverse transition cycle in the pharmaceutical industry. Protein Expr Purif.

[53] Liu W, Hong J, Bevan DR, Zhang YH (2009). Fast identification of thermostable beta-glucosidase mutants on cellobiose by a novel combinatorial selection/screening approach. Biotechnol Bioeng.

[54] Lee HL, Chang CK, Jeng WY, Wang AH, Liang PH (2012). Mutations in the substrate entrance region of beta-glucosidase from *Trichoderma reesei* improve enzyme activity and thermostability. Protein Eng Des Sel.

[55] Pei XQ, Yi ZL, Tang CG, Wu ZL (2011). Three amino acid changes contribute markedly to the thermostability of beta-glucosidase BglC from *Thermobifida fusca*. Bioresour Technol.

[56] Strohl WR (2015). Fusion proteins for half-life extension of biologics as a strategy to make biobetters. BioDrugs.

[57] Liu D, Du K, Feng W (2018). Immobilization of enzymes using a multifunctional fusion polypeptide. Biotechnol Lett.

[58] Conrad U, Plagmann I, Malchow S, Sack M, Floss DM, Kruglov AA, Nedospasov SA, Rose-John S, Scheller J (2011). ELPylated anti-human TNF therapeutic single-domain antibodies for prevention of lethal septic shock. Plant Biotechnol J.

[59] Cho Y, Sagle LB, Iimura S, Zhang Y, Kherb J, Chilkoti A, Cremer PS (2009). Hydrogen bonding of beta-turn structure is stabilized in D(2)O. J Am Chem Soc.

[60] Vogt G, Woell S, Argos P (1997). Protein thermal stability, hydrogen bonds, and ion pairs. J Mol Biol.

[61] Ge X, Yang DSC, Trabbic-Carlson K, Kim B, Chilkoti A, Filipe CDM (2005). Self-cleavable stimulus responsive tags for protein purification without chromatography. J Am Chem Soc.

[62] Banki MR, Feng L, Wood DW (2005). Simple bioseparations using self-cleaving elastin-like polypeptide tags. Nat Methods.

[63] Fong BA, Wood DW (2010). Expression and purification of ELP-intein-tagged target proteins in high cell density *E. coli* fermentation. Microb Cell Fact.

[64] Liu F, Tsai SL, Madan B, Chen W (2012). Engineering a high-affinity scaffold for non-chromatographic protein purification via intein-mediated cleavage. Biotechnol Bioeng.

[65] Zhou Y, Yuan S, Liu Q, Yan D, Wang Y, Gao L (2017). Synchronized purification and immobilization of his-tagged beta-glucosidase via Fe3O4/PMG core/shell magnetic nanoparticles. Sci Rep.

[66] Zhou Y, Yan D, Yuan S, Chen Y, Fletcher EE, Shi H (2018). Selective binding, magnetic separation and purification of histidine-tagged protein using biopolymer magnetic core-shell nanoparticles. Protein Expr Purif.

[67] Sorensen A, Lubeck M, Lubeck PS, Ahring BK (2013). Fungal Beta-glucosidases: a bottleneck in industrial use of lignocellulosic materials. Biomolecules.

