# Supplementary material for: Multifunctional elastin-like polypeptide renders β-glucosidase enzyme phase transition and high stability
Source: Biotechnol Biofuels. 2019 Jun 24;12:157. doi: 10.1186/s13068-019-1497-5 (PMC6589881; doi:10.1186/s13068-019-1497-5)
Supplement: Supplementary file 1 — Additional file 1: Figure S1. SDS-PAGE analysis of BglucH and BglucLEH enzyme. (a): SDS–PAGE analysis of BglucH enzyme and BglucLEH enzyme induce at 37 °C. Lane M, Protein molecular weight marker (Broad); Lane 1, cell lysates of BglucH induced 0 h; Lane 2, cell lysates of BglucH induced 8 h; Lane 3, precipitation lysates containing BglucH; Lane 4, crude cell lysate of BglucH; Lane 5, crude cell lysate of empty pET-28a(+) vector; Lane 6, cell lysates of BglucLEH induced 0 h; Lane 7, cell lysates of BglucLEH induced 8 h; Lane 8: precipitation lysates containing BglucLEH; Lane 9, crude cell lysate of BglucH; The arrows in the figure are the insoluble fractions of the BglucH enzyme and the BglucLEH enzyme. (b): SDS–PAGE analysis of BglucH enzyme and BglucLEH enzyme induced at 25 °C. Lane M, Protein molecular weight marker (Broad); Lane 1, cell lysates of BglucH induced 0 h; Lane 2, cell lysates of BglucH induced 8 h; Lane 3, precipitation lysates containing BglucH; Lane 4, crude cell lysate of BglucH; Lane 5, crude cell lysate of empty pET-28a(+) vector; Lane 6, cell lysates of BglucLEH induced 0 h; Lane 7, cell lysates of BglucLEH induced 8 h; Lane 8: precipitation lysates containing BglucLEH; Lane 9, crude cell lysate of BglucH; The red wireframe in the figure indicates the whole bacteria, sediment and supernatant solution containing BglucH enzyme and BglucLEH enzyme, respectively. Figure S2. SDS–PAGE analysis of BglucH induced at 25 °C and purification by Ni-NTA resin. Lane M, Protein molecular weight marker (Broad); Lane 1, crude cell lysate of cell with empty pET-28a(+) vector; Lane 2, crude cell lysate containing BglucH; Lanes 3–4, purified BglucH by Ni-NTA resin. Figure S3. SDS–PAGE analysis of twice ITC and 400mM imidazole purified BglucLEH. Lane M, Protein molecular weight marker (Broad); Lane 1, crude cell lysate of cell with empty pET-28a(+) vector; Lane 2, crude cell lysate containing BglucLEH; Lanes 3–4, BglucLEH purified after one and two rounds of ITC ope [file 13068_2019_1497_MOESM1_ESM.doc]

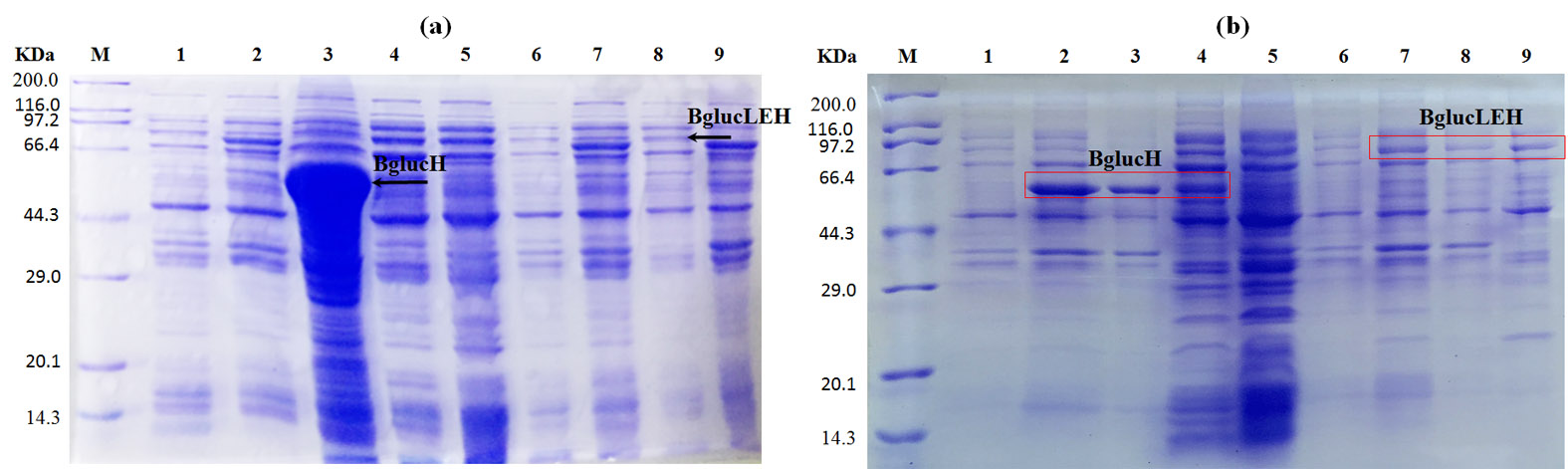


Figure S1. (a): SDS-PAGE analysis of BglucH enzyme and BglucLEH enzyme induce at 37 °C. Lane M, Protein molecular weight marker (Broad); Lane 1, cell lysates of BglucH induced 0 h; Lane 2, cell lysates of BglucH induced 8 h; Lane 3, precipitation lysates containing BglucH; Lane 4, crude cell lysate of BglucH; Lane 5, crude cell lysate of empty pET-28a(+) vector; Lane 6, cell lysates of BglucLEH induced 0 h; Lane 7, cell lysates of BglucLEH induced 8 h; Lane 8: precipitation lysates containing BglucLEH; Lane 9, crude cell lysate of BglucH; The arrows in the figure are the insoluble fractions of the BglucH enzyme and the BglucLEH enzyme. (b): SDS-PAGE analysis of BglucH enzyme and BglucLEH enzyme induce at 25 °C. Lane M, Protein molecular weight marker (Broad); Lane 1, cell lysates of BglucH induced 0 h; Lane 2, cell lysates of BglucH induced 8 h; Lane 3, precipitation lysates containing BglucH; Lane 4, crude cell lysate of BglucH; Lane 5, crude cell lysate of empty pET-28a(+) vector; Lane 6, cell lysates of BglucLEH induced 0 h; Lane 7, cell lysates of BglucLEH induced 8 h; Lane 8: precipitation lysates containing BglucLEH; Lane 9, crude cell lysate of BglucH; The red wireframe in the figure indicates the whole bacteria, sediment and supernatant solution containing BglucH enzyme and BglucLEH enzyme, respectively.


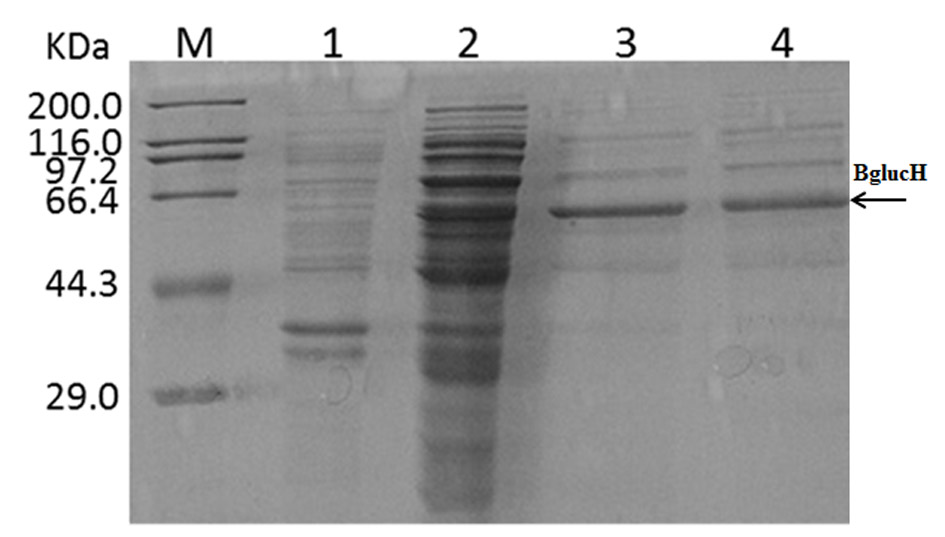


Figure S2. SDS-PAGE analysis of BglucH induced at 25 °C and purification by Ni-NTA resin. Lane M, Protein molecular weight marker (Broad); Lane 1, crude cell lysate of cell with empty pET-28a(+) vector; Lane 2, crude cell lysate containing BglucH; Lane 3-4, purified BglucH by Ni-NTA resin.


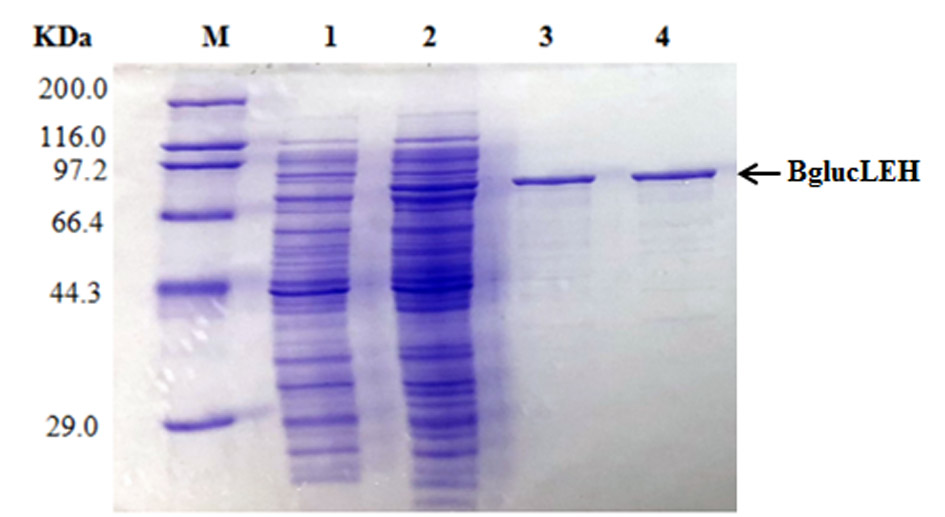


Figure S3. SDS-PAGE analysis of twice ITC and 400mM imidazole purified BglucLEH. Lane M, Protein molecular weight marker (Broad); Lane 1, crude cell lysate of cell with empty pET-28a(+) vector; Lane 2, crude cell lysate containing BglucLEH; Lane 3-4, BglucLEH purified after one and two round of ITC operation using 0.5 M of (NH_4_)_2_SO_4_.


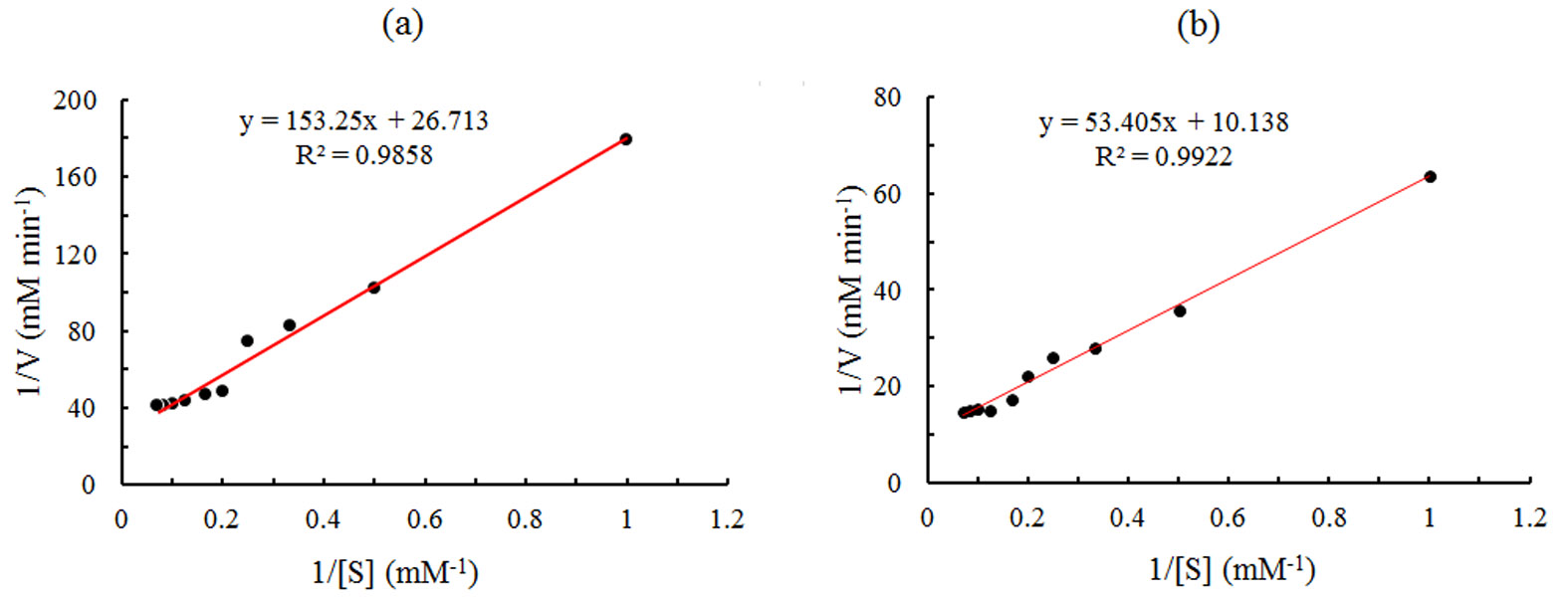


Figure S4. Lineweaver-Burk plots for (a) BglucLEH, (b) BglucLEH.
